# TMH: Two-Tower Multi-Head Attention neural network for CTR prediction

**DOI:** 10.1371/journal.pone.0295440

**Published:** 2024-03-15

**Authors:** Zijian An, Inwhee Joe

**Affiliations:** Department of Computer Science, Hanyang University, Seoul, South Korea; UNITEN: Universiti Tenaga Nasional, MALAYSIA

## Abstract

Click-through rate (CTR) prediction is a term used to predict the probability of a user clicking on an ad or item and has become a popular research area in advertising. As the volume of Internet data increases, the labor costs of traditional feature engineering continue to rise. To reduce the dependence on feature interactions, this paper proposes a fusion model that combines explicit and implicit feature interactions, called the Two-Tower Multi-Head Attention Neural Network (TMH) approach. The model integrates multiple components such as multi-head attention, residual network, and deep neural networks into an end-to-end model that automatically obtains vector-level combinations of explicit and implicit features to predict click-through rates through higher-order explicit and implicit interactions. We evaluated the effectiveness of TMH in CTR prediction through numerous experiments using three real datasets. The results demonstrate that our proposed method not only outperforms existing prediction methods but also offers good interpretability.

## 1 Introduction

Click-through rate (CTR) prediction, which measures the likelihood of a user clicking on an ad or item, is a metric used by companies to gauge the effectiveness of digital advertising [1, 2]. The application of CTR is not limited to the internet. However, the vast amount of online information can lead to users becoming lost in the sea of data and struggling to find the content that suits them. The ability to recommend more relevant ads to users can improve user satisfaction and increase ad clicks by 1% [3–5], thereby adding tens of millions of dollars to a company’s profits. Calculating a user’s click-through rate using their attributes and historical information can help effectively deliver valuable information to users amidst information overload. From collaborative filtering [6] years ago to today’s machine learning and deep learning-based CTR models, where feature interactions play a central role in CTR prediction research. Unlike continuous features found in images and audio, real-world features are mostly multi-domain category features. For example, the category features in the three domains of the MovieLens-1 dataset: (1) Genre = {Romance, Drama, Comedy, …}, (2) Occupation = {doctor, farmer, scientist,…}, (3) Gender = {M, F}. Third-order features were obtained by manual extraction of: (Gender = M, Age = 25-34, Occupation = doctor, Genre = Drama). In a recommendation system, getting good-quality features requires data scientists to spend a lot of time and money exploring the underlying information in the data and extracting meaningful feature interactions [7]. And the large number of raw features makes it impossible to manually extract all feature interactions [8, 9]. Therefore, automatic learning of interaction features is an important challenge.

In the past, algorithmic researchers have proposed several CTR prediction models as a binary classification problem. Logistic regression (LR) [10] models first-order interactions via linear models to obtain first-order features. Factorization Machine (FM) [11] is a second-order feature interaction model built by learning from the inner product between hidden vectors. Field-aware factorization machines (FFM) [12] introduce field-aware potential vectors to capture feature interactions. These FM-based models can only simulate second-order interactions, and linear modeling limits their representativeness. Recently, many deep learning-based models have been proposed to learn higher-order feature interactions by concatenating field embedding vectors and feeding them into deep neural networks (DNNs). Due to its powerful feature representation learning capability, feature interactions in deep learning-based feature fusion models can be in to different ways: implicit feature interactions and explicit feature interactions [13]. Factorization Machine Supported Neural Networks (FNN) [14] propose a model that combines the advantages of FM and DNN to learn higher-order feature interactions implicit in the data, using pre-trained FM embeddings that are fed into DNN layers. Product-based Neural Network (PNN) [15] also proposes a product-based neural network that introduces a product layer between the embedding and DNN layers without relying on pre-trained FM. The DeepFM [16] model achieves simultaneous acquisition of a combination of low and high-order features by introducing a hybrid architecture that includes both shallow memory and generalization components. However, this hybrid architecture uses implicit feature interaction for higher-order features and the model does not have strong memory and generalization capabilities. The Deep &Cross Network (DCN) [17] model learns explicit higher-order feature interactions. The advantage of the model is that it is lightweight and efficient, but the disadvantage is that the final output of the model is expressed as a special kind of vector expansion, while its feature interactions still occur at the element level, and the final output is somewhat limited. The output of each layer of CrossNet is a scalar multiple of the input vector, and this form limits the expressiveness of the model to some extent. FNN, PNN, DeepFM [14–16], these models have the disadvantage of learning by implicit feature interaction, with unknown feature shapes and lack of interpretability. Also, feature interactions are at the bit level rather than between vector-level. Although it can incorporate DNNs for more detailed learning, it increases the risk of overfitting and loses some generalization ability.

In this paper, we introduce the TMH deep fusion model, which utilizes a two-tower multi-head attention network to facilitate automatic learning of higher-order explicit and implicit feature interactions, occurring at both the vector and bit levels. The method is applied to categorical and numerical input features. Specifically, label encoding is used to convert high-dimensional categorical and numerical raw features into numerical variables. Feeding the feature vectors into the embedding layer embeds both categorical and numerical feature vectors into a low-dimensional space. This reduces the dimensionality of the input features, enabling interactions between different types of features. Next, we introduce a higher-order explicit interaction layer designed to facilitate interactions between different features. In each interaction layer, every feature can interact with all others through a multi-head attention mechanism, automatically detecting relevant features and generating meaningful higher-order features. Furthermore, multi-head attention maps feature to multiple subspaces, capturing diverse feature interactions. We introduce residual network connectivity to the interaction layer, enabling the combination of various feature combinations in different orders. It not only captures the correlation and importance of feature weights but also provides a reliable interpretation. Additionally, higher-order implicit interaction layers further enhance feature interactions. Through multiple layers of non-linear transformations and feature extraction, the network progressively captures complex higher-order features and interactions. Each network layer can combine and transform features from previous layers to form a higher-order feature representation. Finally, predictive accuracy is determined using dot product or cosine similarity methods. In summary, our proposed TMH model excels in modeling complex feature interactions in an explicit and implicit manner. It offers flexibility, interpretability, and the ability to capture higher-order features and complex feature interactions. Additionally, it demonstrates strong memorability and generalization, contributing to enhanced predictive accuracy.

The main contributions of this paper are as follows:

1) We propose that the research will combine explicit and implicit automatic learning of higher-order feature interactions to explore how their feature interactions work while discovering models with good interpretability.
2) The multi-head attention mechanism and residual networks designed in the TMH model not only learn higher-order feature interactions explicitly and implicitly, but also capture complex correlations in the input data effectively. With this design, experimental results show that the model not only explicitly obtains higher-order feature combinations, but also significantly improves the accuracy of the model.
3) Extensive experiments on three datasets—Criteo, Avazu and MovieLens-1M have shown that our TMH model significantly outperforms several other state-of-the-art models.

This paper is organized as follows. In Section 2, we discuss related work and the detailed structure of the TMH model presented in Section 3. Then, in Section 4, we present experimental results and a detailed analysis. Finally, in Section 5, we summarise the model presented in this paper and present directions for future work.

## 2 Related work

There are two main approaches to traditional CTR prediction models. Logistic regression(LR) [10] is a simple and effective linear approach that models only first-order interactions on linear combinations of the original features and can capture the linear relationship between features and binary outcome probabilities. The other is the factorization machine (FM) [11], which uses factorization techniques to model second-order feature interactions but cannot fully capture the higher-order interactions present in the data. FM relies on feature engineering to create meaningful interactions between features, requiring domain expertise and time-consuming experimentation. The field-aware factorization machines (FFM) [12] introduce field-aware potential vectors to capture feature interactions, but these fm-based models can only simulate second-order interactions, and linear modeling limits their representativeness. Attentional Factorization Machines (AFM) [18] is based on FM and takes into account the weights of different second-order feature interactions by adding attentional mechanisms to each crossover result based on implicit second-order vector crossovers. These methods can only model lower-order feature interactions. This is not sufficient.

In recent years, deep neural networks (DNNs) have been successful in areas such as computer vision [19]and natural language processing [20]. Because of their deeper structure and non-linear activation functions, researchers have begun to use them to learn higher-order feature interactions. Higher-Order Factorization Machine (HOFM) [21]model differs from traditional FM models by introducing higher-order feature interactions and uses tensor factorization techniques to efficiently handle higher-order interactions, allowing it to capture interactions involving three or more features, thus more accurately representing complex relationships in the data.

Factorization-machine-supported Neural Networks (FNN) [14]model uses pre-trained FM embeddings to be fed into the DNN layers, thus speeding up model convergence. Product Neural Network (PNN) [15]model introduces a product layer between the embedding layer and the DNN layer, i.e. the inner and outer product layers. To learn vector-wise interactions and bit-wise interactions, respectively, to improve the feature crossover ability. Wide&Deep [9]model uses the wide part to enhance the interaction of low-order features and the deep part to enhance the interaction of high-order features. The model achieves simultaneous acquisition of both low and high-order features, with the drawback that the wide part requires manual selection of feature combinations. DeepFM [16] model replaces the Wide part with FM on top of the Wide&Deep model, enhancing the feature crossover capability of the Wide part. The above model simulates implicit higher-order feature interactions by using DNNs. Its feature form is unknown and lacks interpretability. Deep&Cross [17]model replaces the Wide part of the Wide&Deep model with a Cross network to solve the problem of manual combinations by explicitly exploring the interaction of features on a level-by-level basis in a recursive manner.

In contemporary machine learning models, the inclusion of attention mechanisms has become increasingly prevalent. These mechanisms excel at discerning the significance of individual features. Attention mechanisms assign varying degrees of importance to different features. Features that exert a more substantial influence on prediction results are given greater weight, resulting in improved model performance. AutoInt [22] model uses the transform architecture to learn the order of different feature combinations of input features to obtain higher-order feature combinations with weight information, with strong interpretability. FiBiNET [23] model can efficiently capture the complex interactions between low and high-order features through a bilinear convergence layer. This allows the model to better understand the correlation between features, thus improving the accuracy of the recommendation system. The xDeepInt [24] model uses polynomial interaction layers to recursively learn higher-order vector-level and bit-level interactions without the need for jointly trained DNNs and non-linear activation functions. The HoINT [25] model adopts a multi-head attention network with residual connections to explicitly learn high-order feature interactions. Using bilinear functions combined with DNNs to model High-order feature interactions implicitly. This model can actively identify important feature interactions that have good interpretability without artificial feature engineering. DIFM [26] model based on the IFM [27], model adds a transformer to the network, further studies input-aware factors at the vector-wise level. The DTM [28] model automatically obtains high-order and low-order feature combinations in a matrix-level manner based on input features. Using a multi-head attention network, it can achieve bit-level explicit feature combination acquisition. MOFM [29] model interacts with the original features at LR, preserving the memory function of the original features. FM models the second-order interaction features of the embedding vectors. The multi-head attention network with residual connections is added to model the higher-order interactions of the embedding vectors.

## 3 Methods

**Fig 1:**
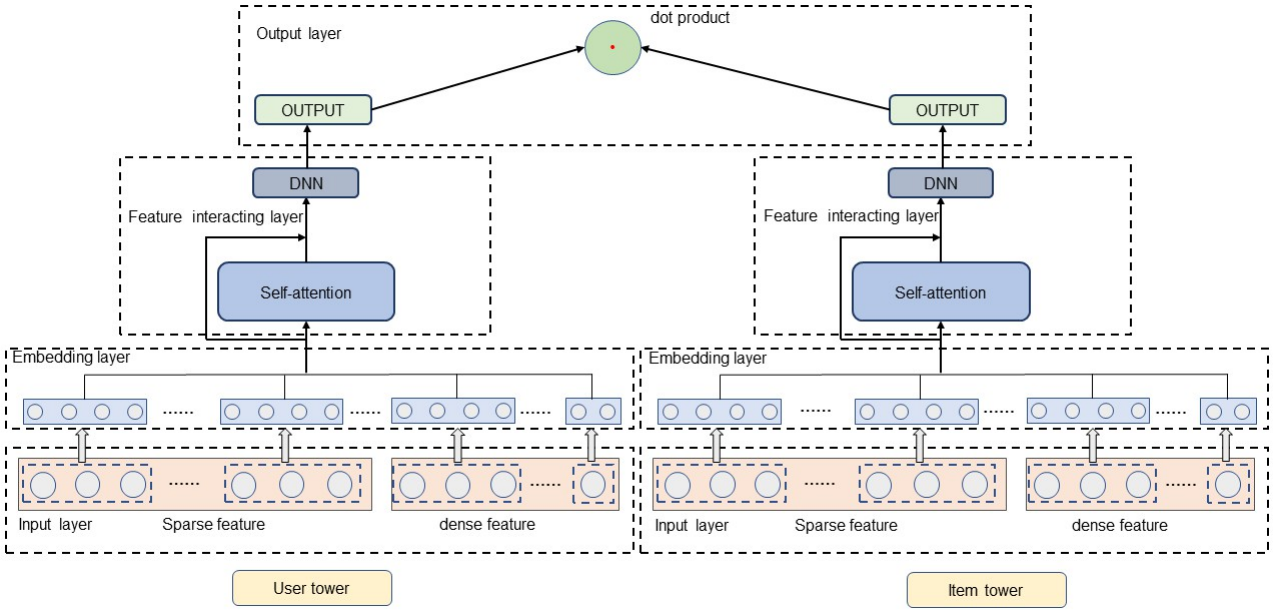
The structure diagram of TMH.

In this section, we introduce the TMH method, which automatically learns features for predicting click-through rates by capturing higher-order explicit and implicit interactions. As shown in Fig 1. The TMH method is carried out in two parts: the user tower and item tower. Initially, the model takes both the numerical and categorical features of users and items as inputs, which are then processed through the user and item towers. In the embedding layer, categorical variables are transformed into dense numerical representations of specified dimensions. This enables interactions between categorical and numerical features. The feature interaction layer is realized as a multi-head attention neural network. In each interaction layer, higher-order features are merged using an attention mechanism. Utilizing a multi-head attention mechanism, features can be assigned to different subspaces, focusing on various types of combined features. It will be possible to model the combination of features in different orders. In the interaction layer, feature vectors maintain the same dimensions in both the user tower and the item tower. Feature information is then learned through a deep neural network to capture higher-order implicit interactions. Finally, we estimate the click-through rate using the dot product approach. We will provide a detailed description of our proposed method.

### 3.1 Input layer

Where *j* is the number of total feature fields, *M*_*i*_ is the i-th feature representation. *M*_*j*_ is a label encoding vector if the *i* field is categorical. If the field *M*_*j*_ is a numeric field, then is a scalar value. The user and item features are entered from the user and item towers respectively.

$$\begin{array}{c} M=[M_{1};M_{2};\ldots ;M_{j}] \end{array}$$ (1)

### 3.2 Embedding layer

The feature embedding layer is the second layer of the user and item tower. The representation of category features is usually sparse and high-dimensional. At this layer, we map categorical variables to dense numerical representations of specified dimensions and convert them into dense vectors. These vectors capture the semantic relationships and similarities between the categorical variables. We use a low-dimensional vector to represent each categorical feature as a low-dimensional space. Where *V*_*i*_ is an embedding vector for field *i*, and *M*_*i*_ is an label vector. The categorical features are expressed as formulas (2). To allow for the interaction between categorical and numerical features, the numerical features are expressed as formulas (3). Where *V*_*j*_ is an embedding vector for field m, and *M*_*j*_ is a scalar value. Where *E*_*i*_, *E*_*j*_ ∈ *R*^*d*^ represents the embedding of one field, *d* indicates embedding dimension. Next, the processed embedding vectors are concatenated *E*_*m*_ as input to the next layer.

$$\begin{array}{c} E_{i}=V_{i}M_{i} \end{array}$$ (2)

$$\begin{array}{c} E_{j}=V_{j}M_{j} \end{array}$$ (3)

$$\begin{array}{c} E_{m}=concat(E_{1},E_{2},\ldots ,E_{i};E_{j}) \end{array}$$ (4)

### 3.3 Feature interacting layer

To implement explicit higher-order combinations and to determine which combinations of features are meaningful, we use a multi-head attention mechanism [30]. In an attention space *h*, a query and a set of key-value pairs are mapped to an output, under the particular attention head *h*, define three different matrices $W_{Query}^{\left(h\right)}$, $W_{Value}^{\left(h\right)}$, $W_{Key}^{\left(h\right)}$. Multiply *Em* by the three matrices to obtain three vector representations. Where $W_{Query}^{\left(h\right)},W_{Value}^{\left(h\right)},W_{Key}^{\left(h\right)}\in R^{d^{\prime }\times d}$, $R^{d^{\prime }}$ indicates the space dimension to which it is mapped. $R^{d}$ denotes the original embedding space dimension. Three particular vectors are represented as follows formulas (5), formulas (6), and formulas (7):

$$\begin{array}{c} E_{m}^{h:Query}=W_{Query}^{\left(h\right)}E_{m} \end{array}$$ (5)

$$\begin{array}{c} E_{m}^{h:Value}=W_{Value}^{\left(h\right)}E_{m} \end{array}$$ (6)

$$\begin{array}{c} E_{m}^{h:Key}=W_{Key}^{\left(h\right)}E_{m} \end{array}$$ (7)

Using feature as an example, we next explain how to identify multiple meaningful higher-order features involving features $E_{m}^{h:Query}$, $E_{m}^{h:Value}$, $E_{m}^{h:Key}$. We first define the similarity between the feature $E_{m}^{h:Query}$ and the feature $E_{m}^{h:Key}$ under an attention head *h* formulas (8). In this paper, using the inner product can represent the degree of similarity between two vectors. Next, the *Attention*(*φ*^(*h*)^) is normalized in the last dimension using the S function to normalize the similarity score to between [0-1], and then the weight information formulas (9) is obtained based on the similarity.

$$\begin{array}{c} \varphi^{\left(h\right)}=<E_{m}^{h:Query},E_{m}^{{\left(h:Key\right)}^{T}}> \end{array}$$ (8)

$$\begin{array}{c} Attention(\varphi^{\left(h\right)}=Softmax(\varphi^{\left(h\right)}) \end{array}$$ (9)

Based on the obtained weight information, the new features of feature m can be obtained by weighted summation with $E_{m}^{h:Value}$ calculation. Where $e_{m}^{\left(h\right)}\in R^{\prime }$ represents an association of feature $E_{m}^{h:Value}$ and its correlated features. This is a feature obtained in a subspace. This approach uses a multi-head attention subspace to represent the input data from a different perspective or dimension. See formulas (10).

$$\begin{array}{c} e_{m}^{\left(h\right)}=Attention(\varphi^{\left(h\right)},E_{m}^{h:Value}) \end{array}$$ (10)

Assuming that there are *h* attention subspaces, the results generated in each attention subspace are stitched together to produce the final result as in formulas (11). Where *h* means the number of total heads. Next, to move to the high-dimensional implicit feature interaction, we use a layer of residual networks [31] to retain some of the original information as input for the next stage. Where *ReLU* is the nonlinear activation function and $\omega_{res}\in R^{d^{\prime }h\times d}$ is a projection matrix. The output of this part is a set of feature vectors that can be connected to the output of the explicit higher-order feature interaction module. As in formulas (12). The above is the higher dimensional explicit interaction part as the user tower and the item tower.

$$\begin{array}{c} e_{m}^{\left(h\right)}=concat(e_{m}^{\left(1\right)},e_{m}^{\left(2\right)},\ldots ,e_{m}^{\left(h\right)}) \end{array}$$ (11)

$$\begin{array}{c} a^{\left(l\right)}=ReLU(e_{m}^{\left(h\right)}+\omega_{res}E_{m}) \end{array}$$ (12)

The deep neural network facilitates secondary high-dimensional feature interactions by taking the features extracted by the multi-head Attention and the residual network, which retains some original information, and further learns higher-order feature interactions. Each hidden layer is calculated according to formulas (13). Where *δ* is an activation function, *b* denotes the bias, and *w* is the weight.

$$\begin{array}{c} a^{\left(l+1\right)}=\delta (w^{l}a^{l}+b^{l}) \end{array}$$ (13)

### 3.4 Output layer

In the final CTR prediction, the User Tower and Item Tower inputs undergo a dot product to calculate feature similarity, followed by the application of a sigmoid function to determine the user’s click probability.

$$\begin{array}{c} O_{output}=a^{\left(l+1\right)} \end{array}$$ (14)

$$\begin{array}{c} O_{output}=O_{output\_user}⊙O_{output\_item} \end{array}$$ (15)

### 3.5 Training

Our loss function is a cross-entropy loss function, defined as follows:

$$\begin{array}{c} L=-\frac{1}{N}\sum_{i=1}^{N}\left(y_{i}log\left(\hat{y_{i}}\right)+\left(1-y_{i}\right)log\left(1-\hat{y_{i}}\right)\right) \end{array}$$ (16)

Where *N* is the total number of training samples, *i* is the index of the training sample. *y*_*i*_ and $\hat{y_{i}}$ are the true values of user clicks and estimated CTR, respectively. The CTR dataset had an unbalanced ratio of positive to negative samples, which would bias the predictions. To balance the ratio, we randomly selected the same number of positive and negative samples in each batch during training.

### 3.6 Time complexity and space complexity

We set *d* to be the embedding size, *L* to assume a total of *L* layers of the network, *M* is the number of feature fields, and *H* is the number of heads. As far as interacting layers are concerned, the space complexity is *O*(*Ldd*′*H*). And, Forming combined features under one head requires *O*(*Mdd*′+ *M*^2^*Hd*′). We have H heads, time complexity is *O*(*MHd*′(*M*+ *d*).

## 4 Experiment setup and analysis

### 4.1 Dataset

We used three common real-world datasets with the specific parameters shown in Table 1.

**Table 1:** Statistics of evaluation datasets.

Datasets	Samples	Fields	Sparse Features
MovieLens-1M	739012	7	3529
Avazu	40428967	23	1544499
Criteo	45840617	39	998960

MovieLens-1M dataset comprises 1 million ratings from 6,000 users for 4,000 movies. The user’s ID, gender, age, and occupation are used as inputs to the user tower in the model’s input layer, while the movie ID and genre serve as inputs to the item tower. When dealing with a user’s movie rating, ratings below 3 are classified as negative samples, indicating the user’s dislike, while ratings above 3 are categorized as positive samples.

Avazu4 dataset is from a Kaggle challenge for Avazu CTR prediction, where the goal is to predict whether a mobile ad will be clicked. The dataset contains mobile behavior records for 40 million users, indicating whether a user clicked on a mobile ad. It consists of 23 functional fields, covering user data, device functions, ad attributes, and more. The target variable is whether the ad was clicked (1) as a positive sample or not clicked (0) as a negative sample.

Criteo dataset serves as a standard for click-through rate prediction and includes 45 million instances of user clicks on displayed ads. The dataset encompasses 26 categorical and 13 numerical attribute domains, with the target variable indicating whether an ad was clicked (1) as a positive instance or not clicked (0) as a negative instance.

### 4.2 Data preprocessing

First, we normalize the numerical features and encode the categorical features using label encoder. Outlier normalization involves linearly transforming the original data to constrain resulting values within the range of [0—1]. For example, the MovieLens-1M dataset maps rating values [0—5] to between [0—1]. This is achieved by subtracting the minimum value from the variable value and dividing by the difference between the maximum and minimum values. This eliminates the undesirable effects of odd sample data. Presence of odd sample data can increase training time and may lead to convergence issues. Therefore, it’s crucial to normalize the pre-processed data before training.

Secondly, skewed data distributions can lead to undesirable outcomes. To address this, we can use feature engineering techniques involving statistical or mathematical transformations. When dealing with dense intervals, it is beneficial to spread out the values as much as possible, while for non-dense intervals, clustering the values is recommended. Monotonic transformations are commonly used for this purpose as they help stabilize the variance, normalize the distribution, and render the data independent of the distribution’s mean. As follows formulas (17).

$$\begin{array}{c} y=log_{c}^{1+\beta x} \end{array}$$ (17)

*β* is set to 1 and *c* is usually set to the maximum value of the transformed data. Logarithmic transformation increases the range of lower-order dependent variable values and decreases the range of higher-order dependent variable values. This results in a skewed distribution that is as close to normal as possible.

Finally, we randomly select 90% of all samples for training and randomly divide the rest into validation and test sets of equal size.

### 4.3 Evaluation metrics

We use the following two metrics for model evaluation: AUC and Logloss.

AUC(Area Under the ROC Curve) metric assesses a binary classification model’s performance by estimating the probability of a positive instance ranking higher than a randomly chosen negative instance. A higher AUC score indicates superior model performance.

Logloss quantifies the disparity between the predicted and actual label scores for each instance. Model performance improves with lower logloss values. The logloss score measures a model’s capacity to predict an instance’s class probability and is frequently used in multi-class classification problems.

Learning rates at the 0.001 level, lower log loss, or slightly higher AUC are considered important for the CTR prediction task, as also noted in existing work.

### 4.4 Baselines

LR [10]. first-order interaction modeling is performed by linear combination to obtain first-order features.

FM [11] second-order feature interaction model is built by the inner product between the hidden vectors.

AFM [18] Building on FM, incorporate an attention mechanism into each second-order feature interaction, considering the weights of different interactions.

DeepCrossing [32] implicitly learning higher-order feature interactions using residual connections of DNNs.

NFM [33] Second-order interactions are modelled using a DNN, and then second-order combined features are fed into the DNN to model higher-order interactions.

CrossNet (Deep&Cross) [17] is the core of the Deep&Cross model, replacing the Wide part of the Wide&Deep model with the residual connections of the DNN, which attempts to model the interaction of features explicitly by performing an outer product of the tandem feature vectors at the bit level.

Higher Order Factorisation Machine (HOFM) [21] introduces the use of tensor factorization techniques to efficiently handle higher-order interactions, thus enabling the capture of interactions involving three or more features.

CIN (xDeepFM) [34] is the core of the xDeepFM model, performing an outer multiplicative superposition of vectorial feature matrices to capture explicit high-order features.

Autoint [22] combines higher-order features through an attention mechanism and evaluates different combinations using a polytope mechanism, which can simulate combinatorial properties of different orders.

### 4.5 Implementation details

We use TensorFlow [35] to implement our method. A grid search strategy is used to find the optimal hyperparameters. Our method sets the embedding vector dimension to 12 vector dimensions, the batch size to 1024, and the number of attention heads is set to 12. We use Adam to optimize the deep neural network model. In three large datasets to prevent overfitting, we used a grid search to select a dropout rate of 0.5 from {0.1—0.9} as the baseline implementation follows the criteria of model [22]. The depth crossover has four feedforward layers and our model sets the number of hidden units per layer to 1024, 512, 256, 32} as it performs poorly when using three or five neural network layers. Once all the network configurations had been determined, a grid search was performed on the basic method for the best parameters. All deep neural network models were optimized using Adam and run on an NVIDIA 3090 GPU.

### 4.6 Model comparison

This paper compares the TMH model with several existing models. The performance of all models on three public datasets is shown in Table 2. The observation results are as follows.

(1) The experimental results demonstrate that TMH outperforms classical shallow prediction methods such as LR, FM, and AFM in terms of prediction performance. LR can fuse various types of features but is the worst performer among these baselines due to its limited ability to combine features and poor expressiveness. FM offers second-order feature crossover capabilities compared to logistic regression, significantly improving the model’s expressiveness. However, due to the combinatorial explosion problem, extending the model to third-order feature crossover is challenging. Meanwhile, AFM outperforms FM, highlighting the effectiveness of the attention mechanism in handling various interactions and assigning different levels of importance to crossover features.
(2) We note the drawback of certain models that capture feature interactions of higher order. NFM outperforms FM models, which may be due to the inclusion of neural networks instead of second-order hidden vectors in FM, demonstrating the effectiveness of incorporating NFM into DNN. They are not guaranteed to improve FM and AFM. DeepCrossing outperforms NFM. This demonstrates the effectiveness of residual connectivity in CTR prediction. CrossNet solves the problem of manual feature combination in the Wide&Deep model, but the complexity of the cross-network is high, which has an impact on the accuracy of the model.
(3) HOFM significantly outperforms FM on all three datasets. This indicates that third-order feature interaction modeling is beneficial to CTR prediction performance. CIN can jointly train explicit and implicit high-level feature intersections without requiring manual feature engineering, greatly improving model results. AutoInt introduces a transformer to achieve a higher-order explicit interaction between features, with a multi-head attention mechanism in the transformer. In multiple subspaces, features are interacted and then fused according to different relevance strategies, so that each feature ends up with information about the other features, and the importance of the other features is weighted.
(4) Our proposed TMH achieves the best performance of all these methods on three datasets. Compared to the state-of-the-art models, the TMH model has the best performance of all models on all datasets. On the MovieLens-1m dataset, the log loss for the predictive performance metrics is reduced by about 5%. On the Avzau, Criteo dataset, the AUC of TMH is higher than the state-of-the-art model by about 0.12% and 0.08%, respectively. This proves the effectiveness of the model. The method shows a great advantage, which is the automatic learning of the CTR predictions through higher-order explicit interactions and higher-order implicit interactions, which fully demonstrates the effectiveness of the modeling. Compared to CIN, which is able to learn higher-order feature interactions explicitly and implicitly at the same time, the TMH model improves the AUC on the three datasets by 0.12%, 0.15%, and 0.06%, respectively. This confirms that both implicit and explicit learning of higher-order features are key to improving model performance.

**Table 2:** Comparison of the performance of the different methods.

Model Class	Model	Avazu		MovieLens-1M		Criteo	
		Auc	Logloss	Auc	Logloss	Auc	Logloss
First-order	LR	0.7560	0.3964	0.7716	0.4424	0.7820	0.4695
Second-order	FM	0.7706	0.3856	0.8252	0.3998	0.7836	0.4700
	AFM	0.7718	0.3854	0.8227	0.4048	0.7938	0.4584
High-order	DeepCrossing	0.7643	0.3889	0.8448	0.3814	0.8009	0.4513
	NFM	0.7708	0.3864	0.8357	0.3883	0.7957	0.4562
	CrossNet	0.7667	0.3868	0.7968	0.4266	0.7907	0.4591
	CIN	0.7758	0.3829	0.8286	0.4108	0.8009	0.4517
	HOFM	0.7701	0.3854	0.8304	0.4013	0.8005	0.4508
	AutoInt	0.7752	0.3824	**0.8456**	0.3797	0.8061	0.4455
	TMH	**0.7764**	**0.3817**	0.8440	**0.3268**	**0.8069**	**0.4450**

### 4.7 Ablation study

In this paper, ablation experiments using THM are conducted to validate each component in the HoINT model, in order to better understand their relative importance.

(1) No-TMH: Removal of multi-head self-attention network. The model cannot explicitly model higher-order feature interactions.
(2) No-DNN: DNN is removed. The model cannot implicitly learn higher-order feature interactions.

In this set of experiments, one part was removed and the rest was left unchanged. The following results can be derived from Table 3. Removing any component of TMH will lead to a decline in performance. This confirms that each component of the TMH model advanced in this study is crucial for superior performance.

**Table 3:** Different variants of TMH.

Model	Avazu		MovieLens-1M		Criteo	
	Auc	Logloss	Auc	Logloss	Auc	Logloss
TMH	0.7764	0.3817	0.8440	0.3286	0.8069	0.4450
NO-TMH	0.7686	0.3857	0.8159	0.3958	0.8012	0.4495
NO-DNN	0.7708	0.3852	0.8261	0.3386	0.8044	0.4487

As can be seen from the results for the Avazu and Moivelens-1m datasets, the removal of the DNN significantly decreases the AUC and Logloss performance of the model by about 0.6%, 0.5% and 2%, 1%, respectively. For the Criteo dataset, the AUC performance of the model decreased by 0.15% and the logloss performance decreased by 0.3%. The experimental results show that DNN modelling of implicit higher-order feature interactions has a significant impact on the model results.

After the multi-head self-attention network was removed, the model’s AUC performance on the MovieLens-1m dataset dropped significantly, by about 3%. The AUC performance on the other two data sets dropped by about 0.1% and 0.05% respectively. The experimental results show that multi-head self-attention network modelling of explicit higher-order feature interactions has a significant impact on the model results. We can see that TMH outperforms all ablation methods, which justifies the necessity of all these components in our model.

### 4.8 Parameter study

**Fig 2:**
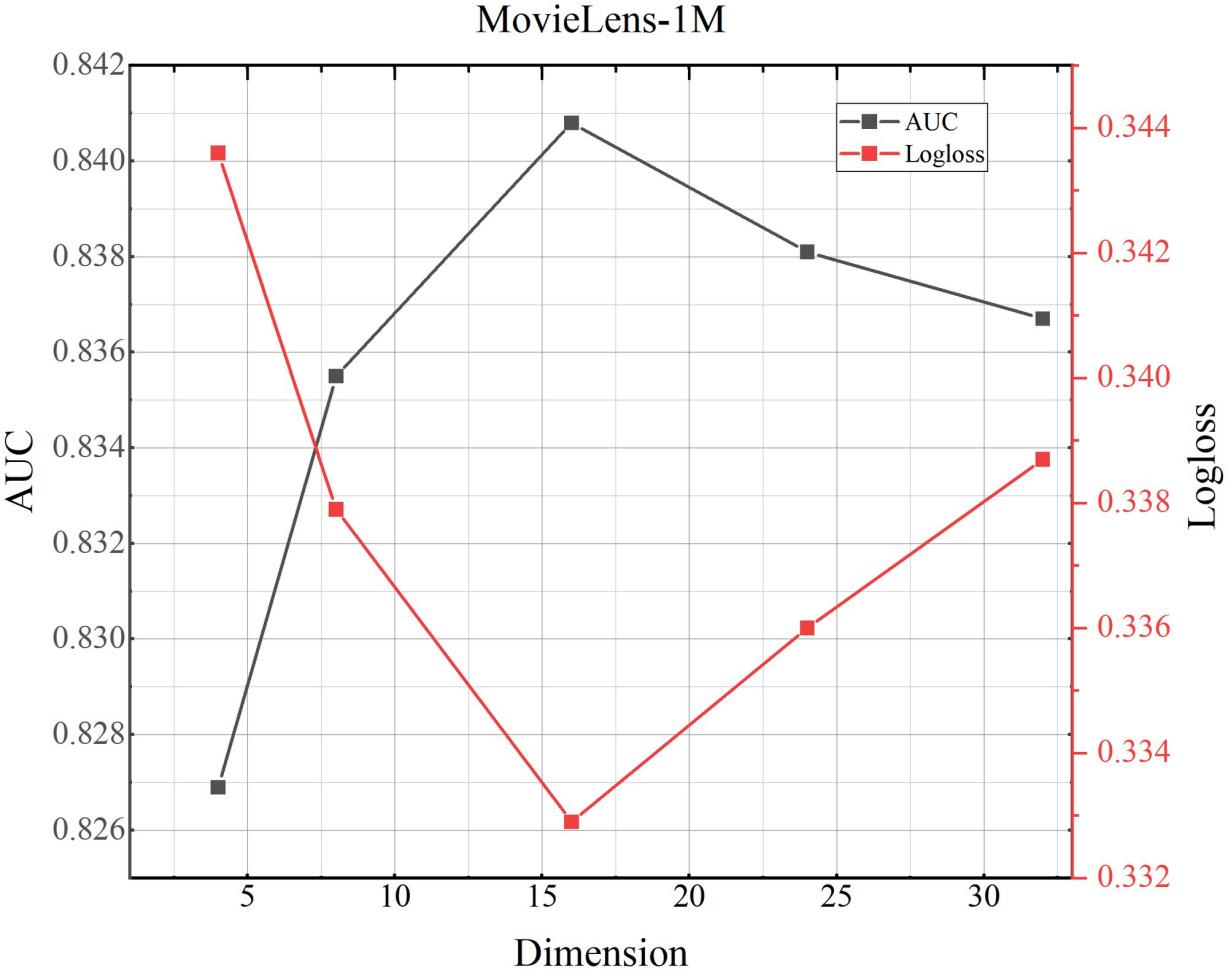
TMH model performance testing the influence of different dimensions on MoiveLens-1M dataset.

**Fig 3:**
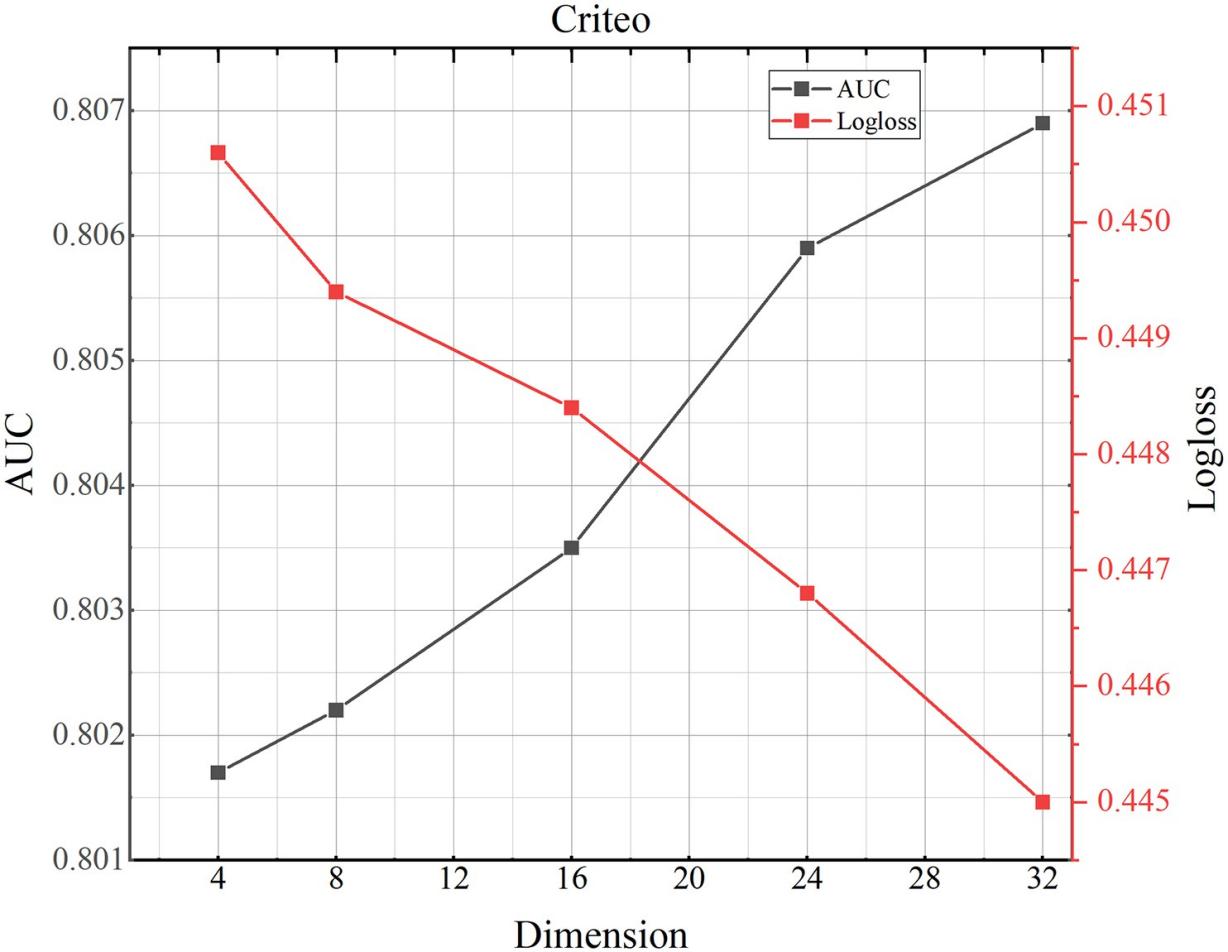
TMH model performance testing the influence of different dimensions on Criteo dataset.

**Fig 4:**
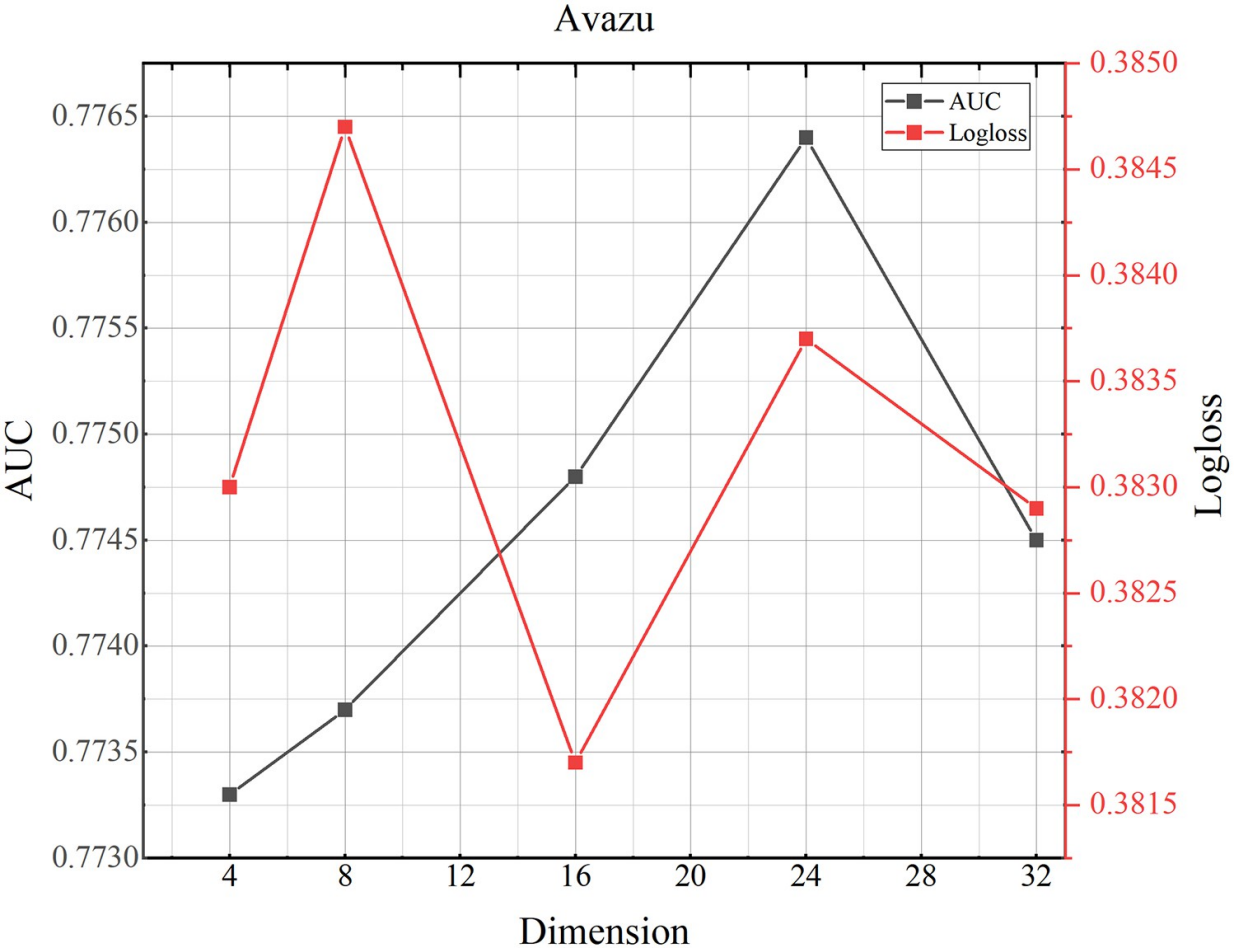
TMH model performance testing the influence of different dimensions on Avazu dataset.

**Fig 5:**
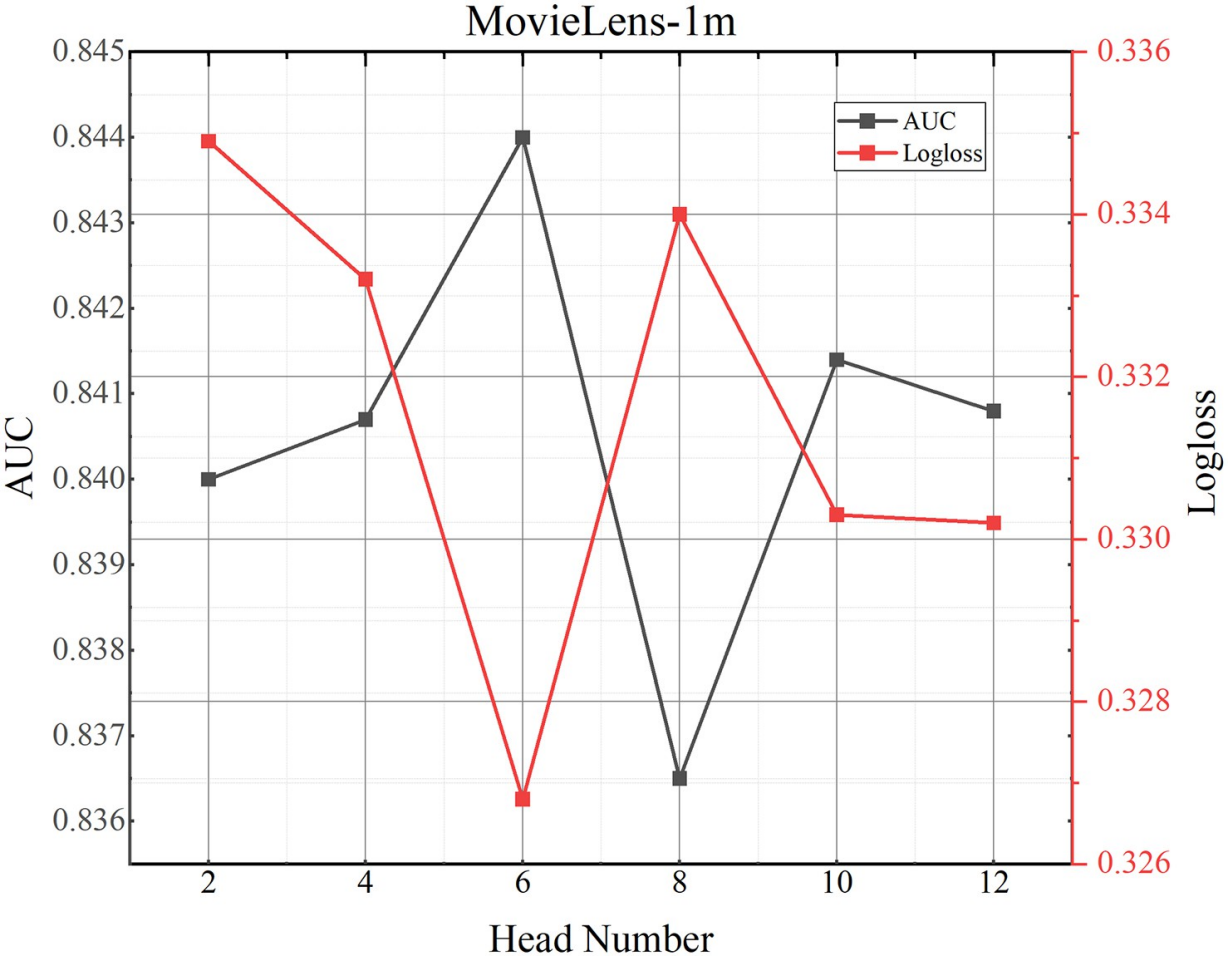
TMH model performance testing the influence of number of the attention heads on MoiveLens-1M dataset.

**Fig 6:**
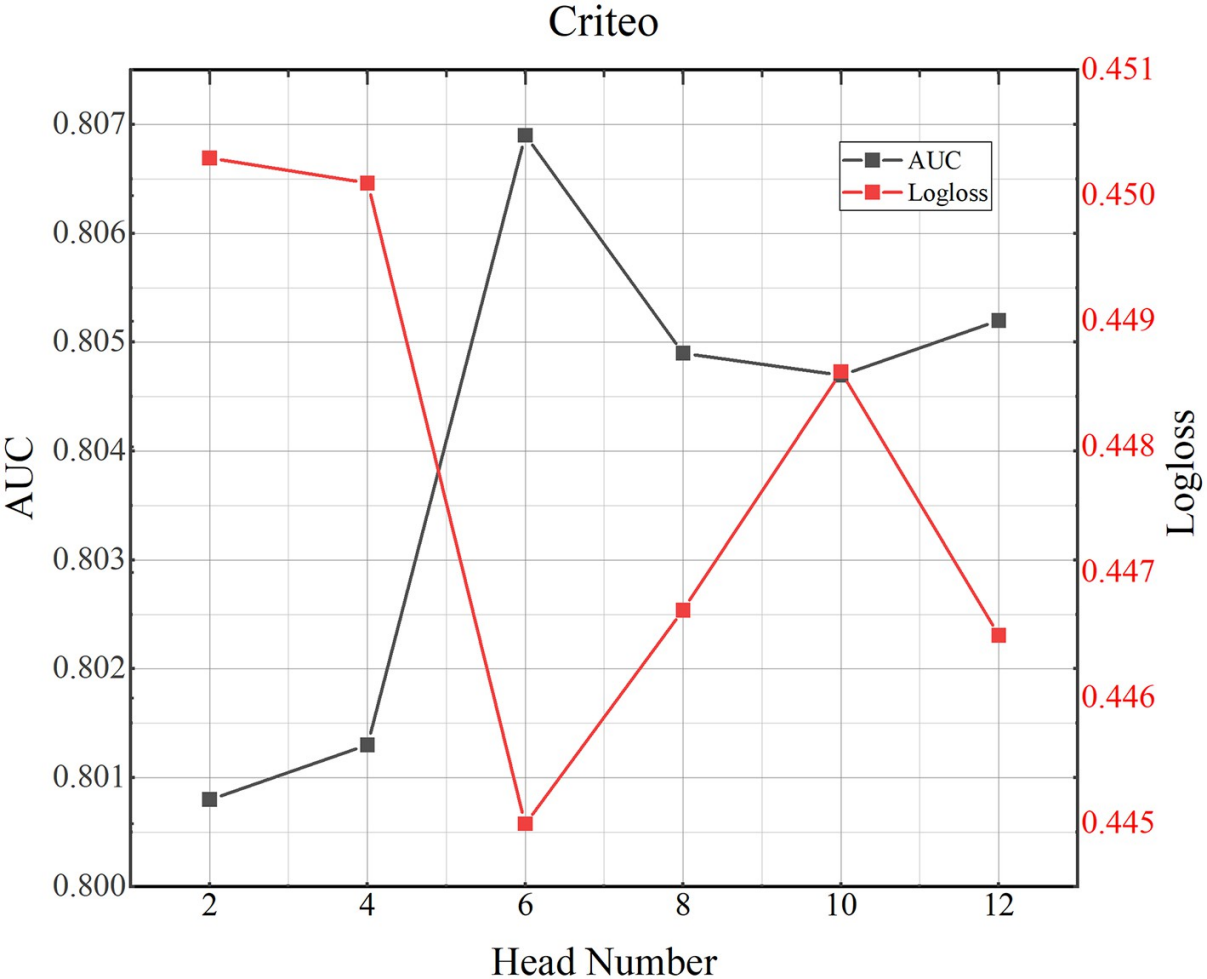
TMH model performance testing the influence of number of the attention heads on Criteo dataset.

**Fig 7:**
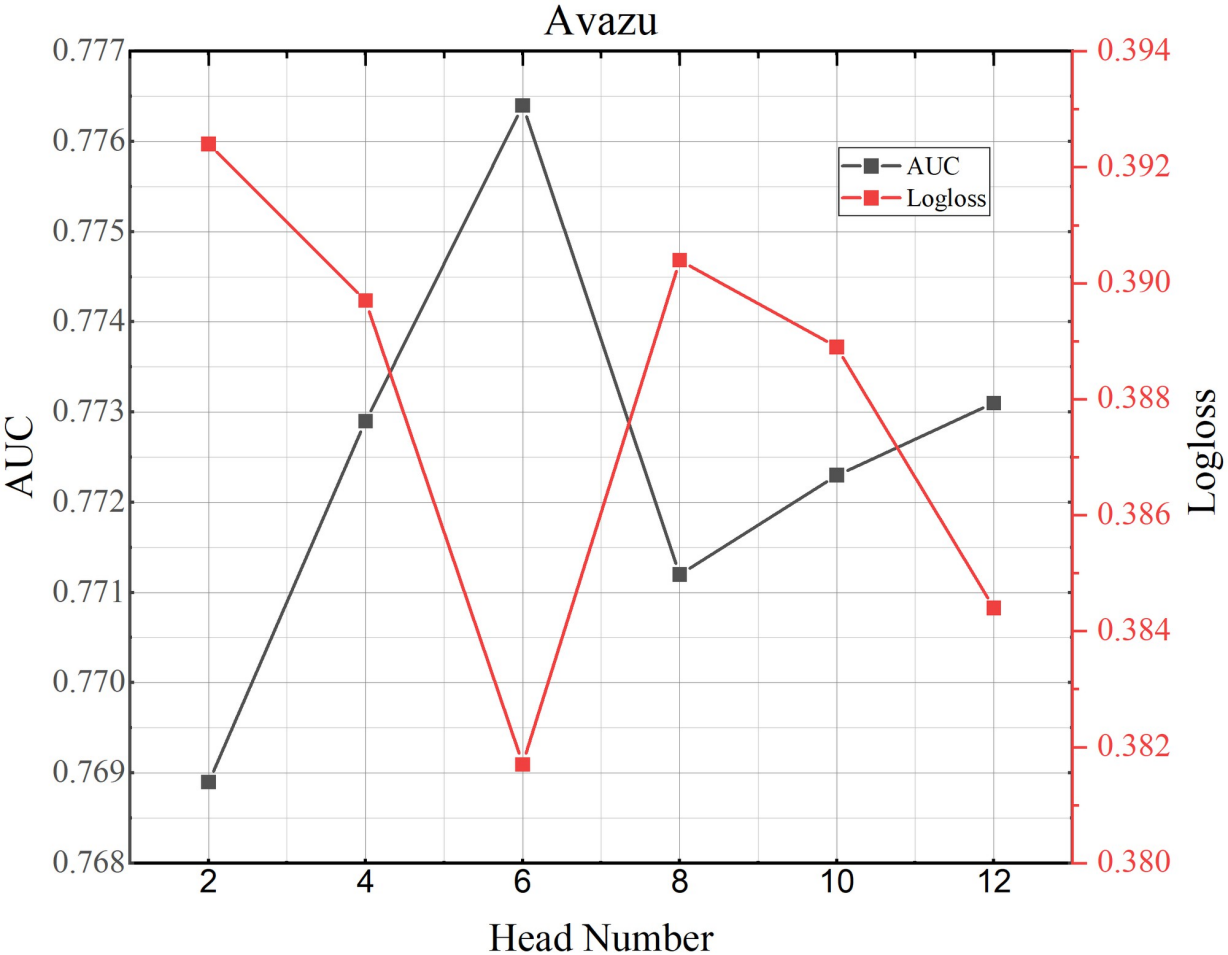
TMH model performance testing the influence of number of the attention heads on Avazu dataset.

**Fig 8:**
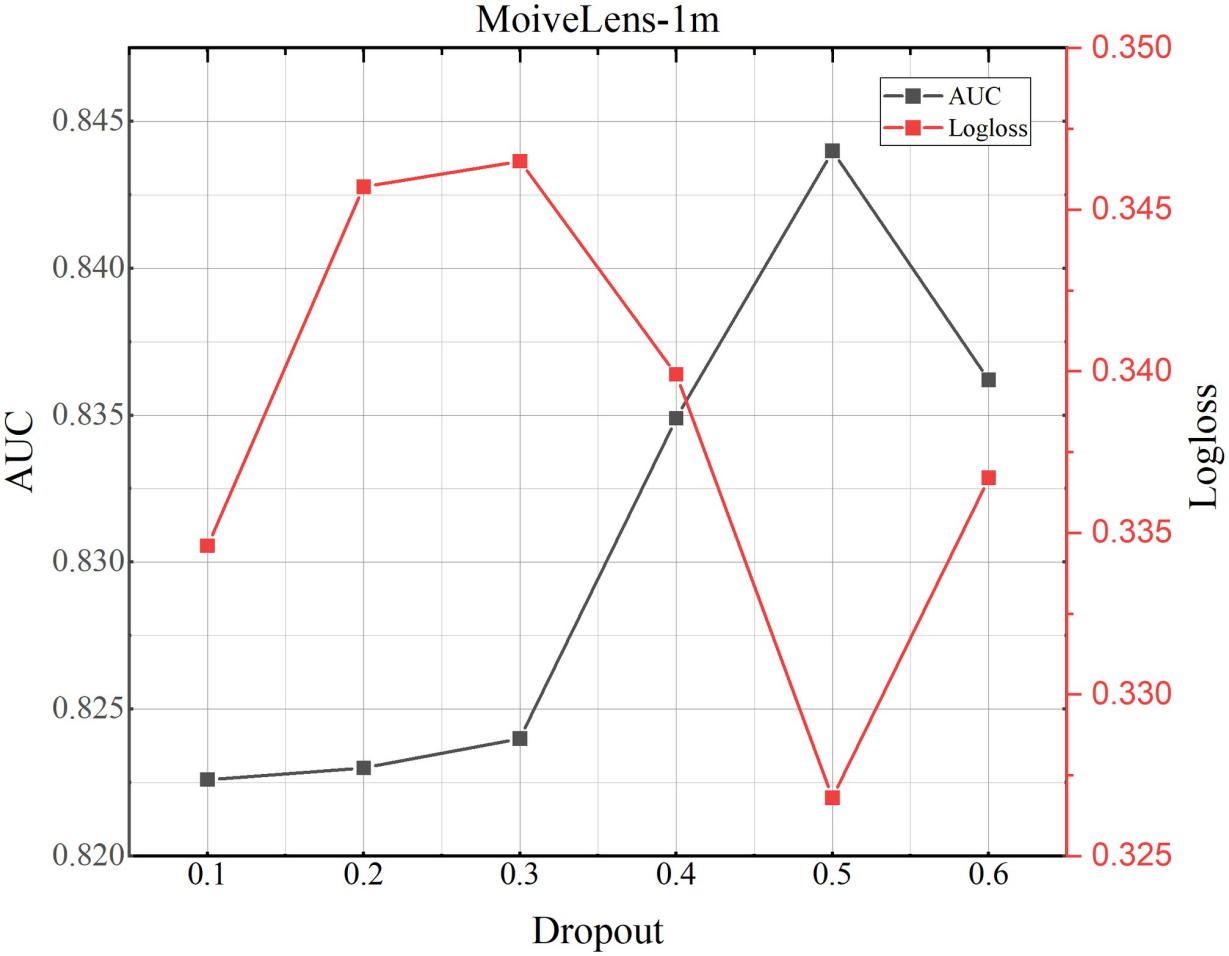
TMH model performance testing the influence of dropout on MoiveLens-1M dataset.

**Fig 9:**
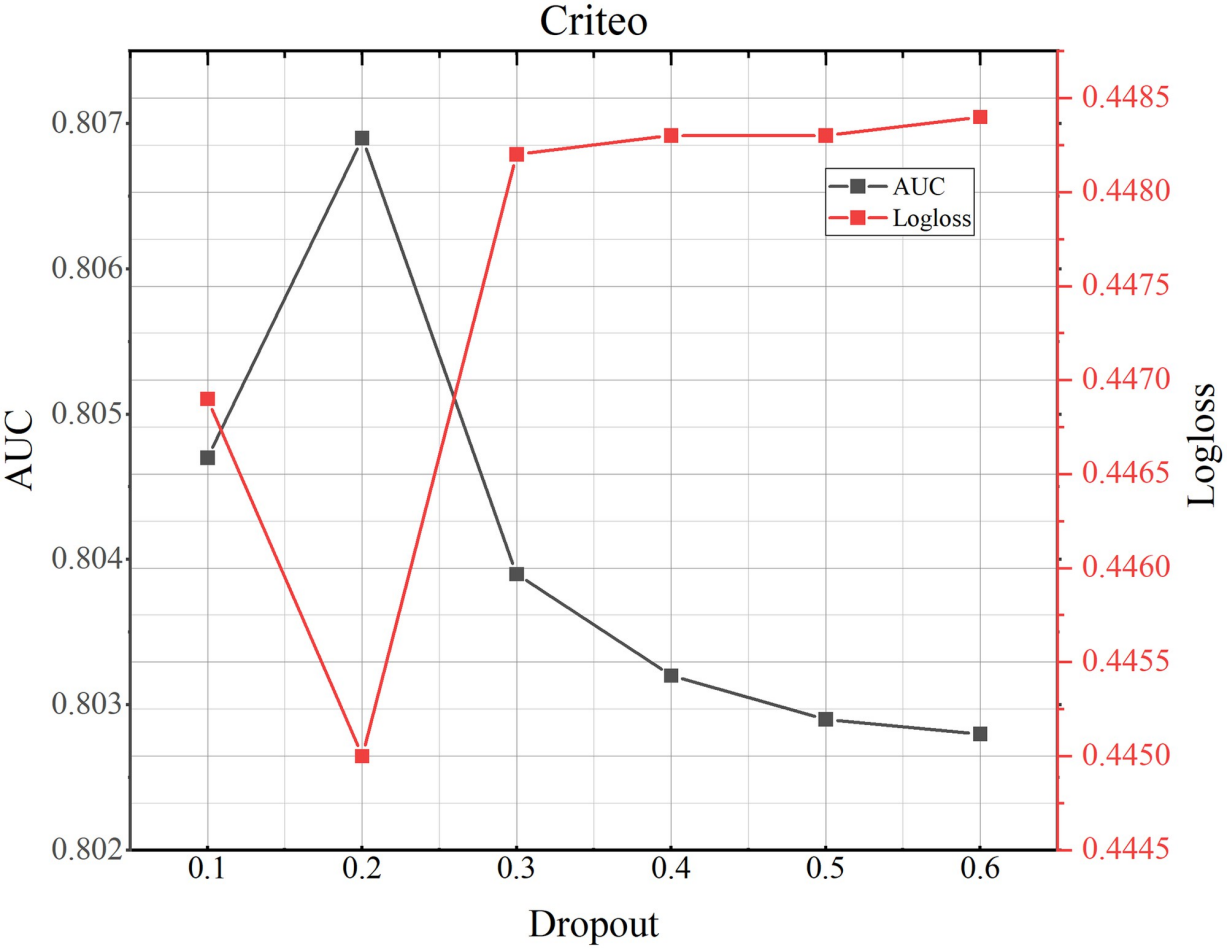
TMH model performance testing the influence of dropout on Criteo dataset.

**Fig 10:**
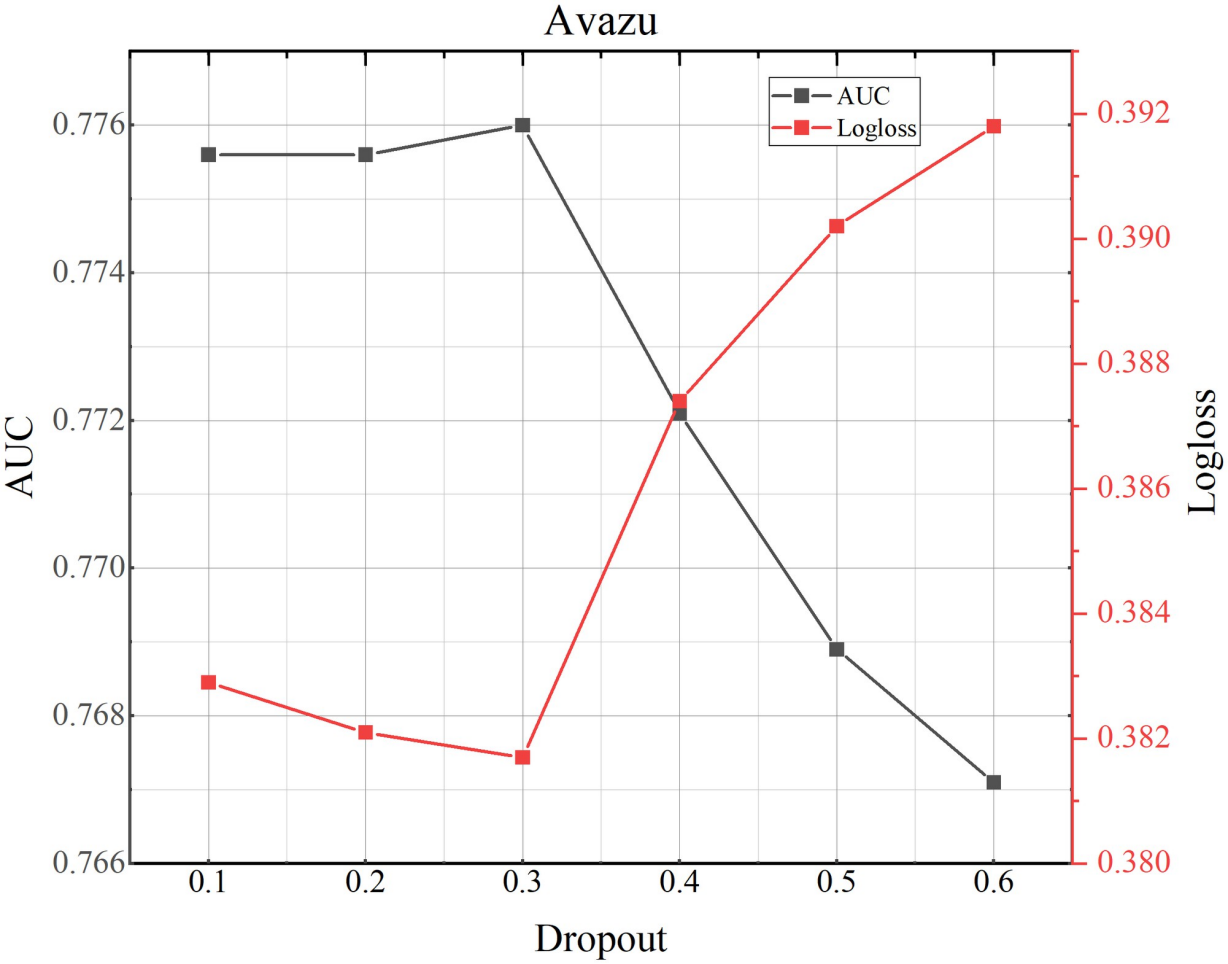
TMH model performance testing the influence of dropout on Avazu dataset.

(1) Influence of different dimensions.
  We investigate how the dimensionality of the domain embedding vectors affects performance. The results for the MovieLens-1M, Criteo, and Avazu datasets are shown in Figs 2–4. As shown for the MovieLens-1Mh and Avazu datasets, we can see that performance increases with increasing dimensionality, as larger dimensional vectors can represent more information. Performance is optimal when the vector size reaches 16h and 24 respectively. Beyond 16h and 24, performance begins to decrease as the states in this dimension already contain enough information that if too many parameters are generated, the model will be over-fitted, resulting in reduced accuracy and increased log loss. Table 1 shows that Criteo has the largest total amount of data, so the corresponding number of parameters becomes larger and the data fits better.
(2) Influence of number of the attention heads
  Figs 5–7 shows the effect of different numbers of attention heads on the TMH model. To some extent, the larger the number of attention heads the more expressive the whole model is. The more it can improve the model for the reasonable allocation of attention weights. As can be seen in Fig 4, as the number of heads increases from 2 to 12, the AUC and Logloss performance of the model first decreases and then increases for the AUC value and Logloss value in Criteo, Avazu and Movielens-1 M datasets. And, when the number of heads is set to 8, there is an up and down fluctuation. We synthesize it and set the number of heads to 6.
(3) Influence of dropout
  The main function of the dropout rate is to prevent overreliance on training data and improve parameter generalization. A small dropout value doesn’t effectively reduce overfitting, while a high value can lead to the loss of important information, affecting recommendations. As shown in Figs 8–10, the AUC and loss of the TMH model decreases and then increases on the Criteo and Avazu datasets. On the contrary, the AUC and loss values of the model show a trend of increasing and then decreasing on the MovieLens-1m dataset. We applied dropout rates of 0.2, 0.3, and 0.5 on the Criteo, Avazu, and MovieLens-1m datasets, respectively.

### 4.9 Model interpretability

**Fig 11:**
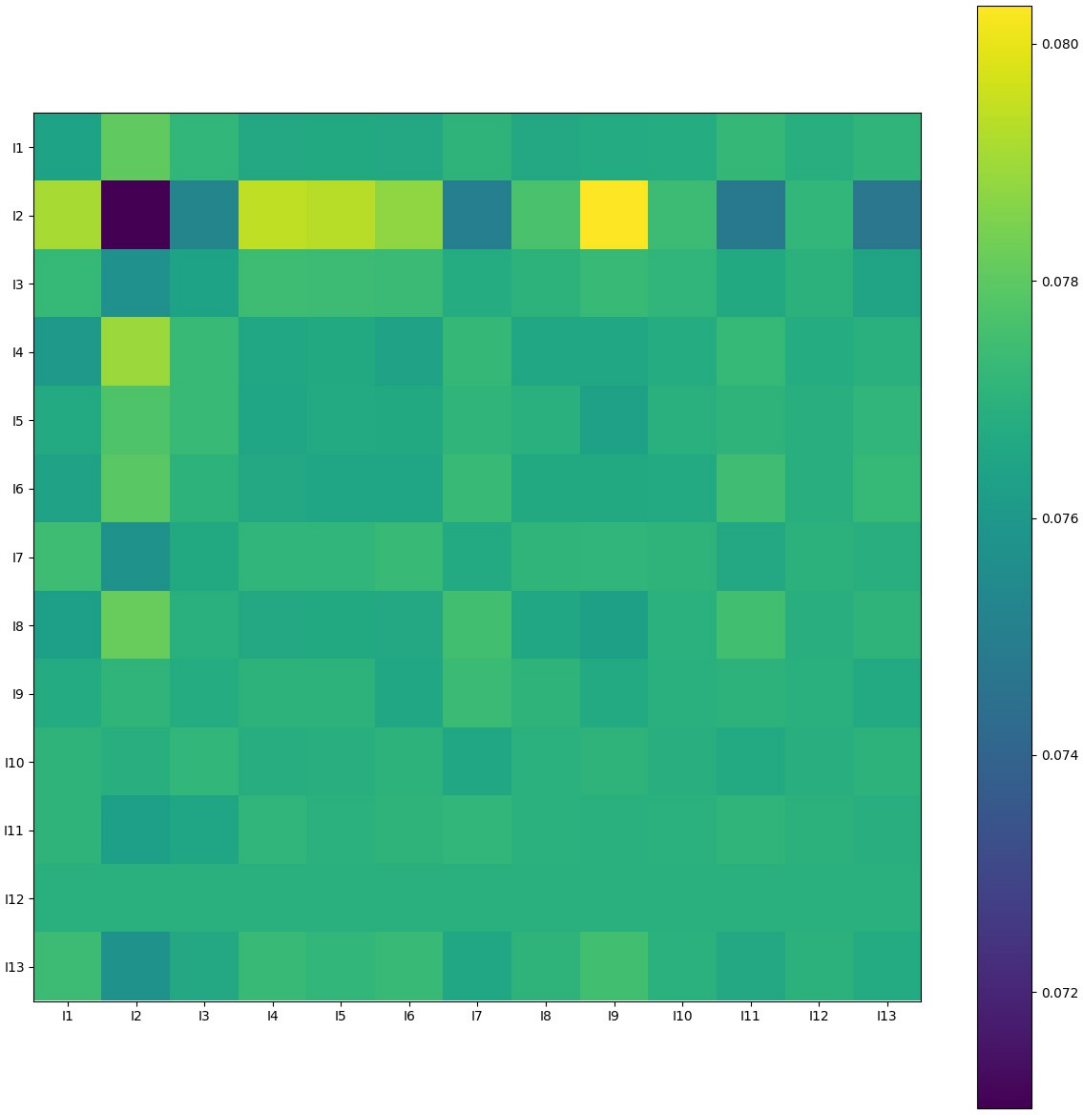
Heat map of attention scores on features I1-I13 on the criteo dataset, which reflects the importance of the relationship between the different feature fields.

**Fig 12:**
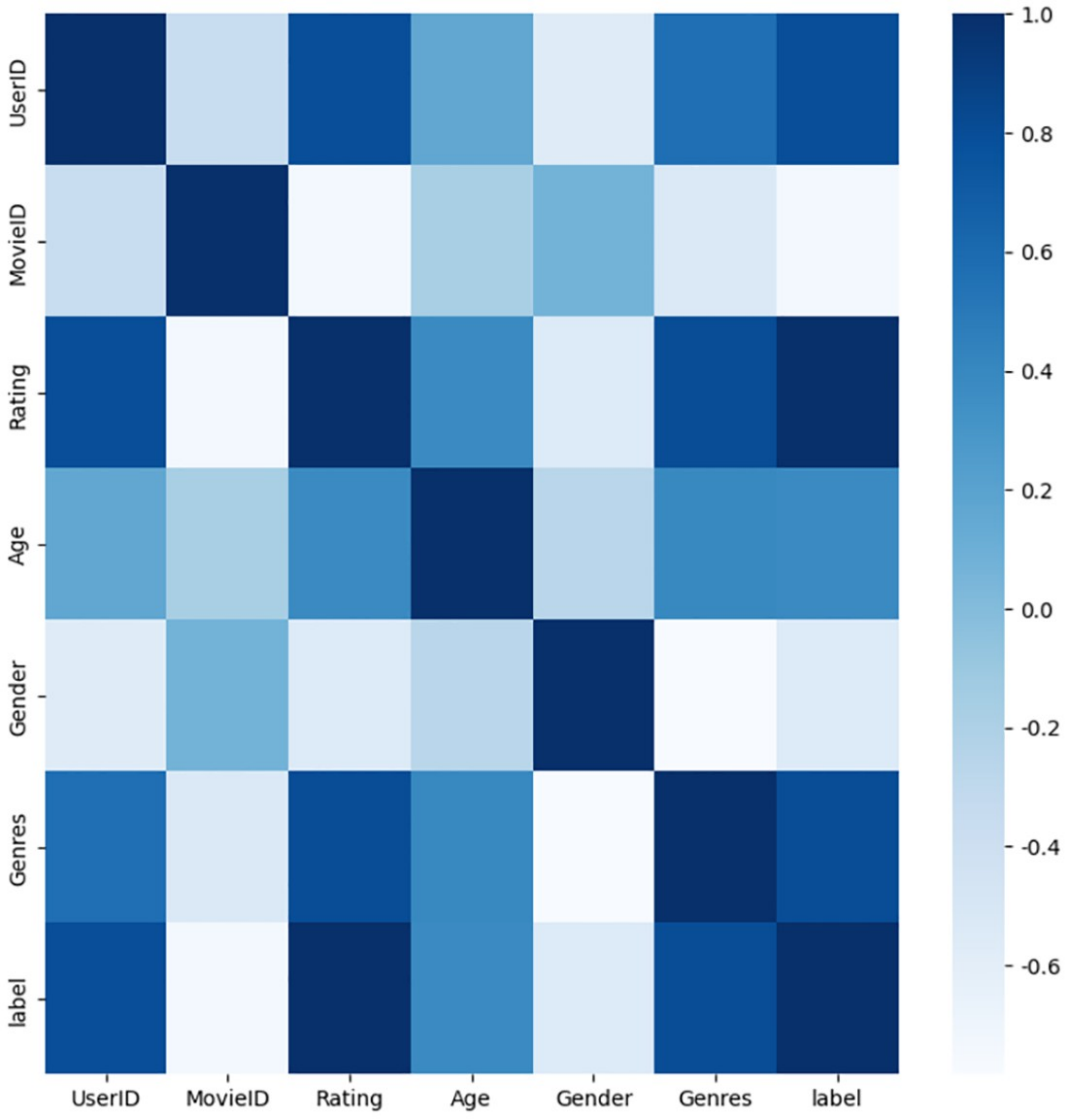
Heat map of attention scores on the MovieLens-1M dataset, which reflects the importance of the relationship between the different feature fields.

(1) Model interpretability on the Criteo dataset
  To illustrate the importance of feature interactions and enhance model interpretability, we will use the Criteo dataset as an example. Fig 11 illustrates the correlation between different domains of the input features. For this analysis, we focused on features within the I1-I13 range in the dataset, which contains some anonymous feature fields. In the heatmap, yellow indicates higher attention, while black represents lower attention. The heatmap reveals that (I2, I2) is marked in black, suggesting that there is lower attention or less available information in this feature region. Conversely, the (I10, I2) region is highlighted in yellow, indicating a higher level of attention. This suggests a correlation between the features in I10 and I2. This correlation between features within the I1-I13 dataset may be indicative of click behavior and its dependence on a key feature field.
(2) Model interpretability on the MoiveLens-1m dataset
Fig 12 displays the correlation between various attribute features in the dataset. This axis represents the feature field (UserID, MoiveID, Rating, Age, Gender, Genres, Label). We can observe that (Gender, Genres), (Rating, Label), (age, Genres), etc. features exhibit strong correlations. (Age, Gender) features are very important. The strong correlation between age and movie genre performance suggests that users of different ages have distinct genre preferences. Additionally, gender plays a significant role in determining movie ratings across various genres, making it a crucial factor in understanding user preferences for movies. In summary, the visualization results underscore the importance of feature interactions, which hold critical implications for feature engineering and model comprehension.

## 5 Conclusion future work

In this research, we have developed a new ctr prediction based on a multi-head attention mechanism, a model that automatically learns features through higher-order explicit interactions and higher-order implicit interactions. Correlations between features are determined by allowing feature-feature interactions to occur in each sub-space of the multi-head attention layer. Secondary higher-order feature interactions are then performed in the DNN layer. We have conducted experiments on three experimental datasets, and the results clearly demonstrate that our proposed method is effective and shows good results in terms of logloss and AUC scores.

Machine learning and deep learning perform very well in prediction tasks in various industries. However, most of the use in industry is based on deterministic baseline models. In the future, we will try to test our proposed model with more industrial data to prove that the model is simple and efficient and remains useful in industry. Secondly, in the text processing and vision area, the TMH model proposed in this paper is used as the base model. Text features and image features are added to the test to build a model for text and image CTR prediction.
